# Identification of heat shock protein70-2 and protamine-1 mRNA, proteins, and analyses of their association with fertility using frozen-thawed sperm in Madura bulls

**DOI:** 10.5713/ab.23.0142

**Published:** 2023-06-26

**Authors:** Zulfi Nur Amrina Rosyada, Berlin Pandapotan Pardede, Ekayanti Mulyawati Kaiin, Ligaya I.T.A Tumbelaka, Dedy Duryadi Solihin, Bambang Purwantara, Mokhamad Fakhrul Ulum

**Affiliations:** 1 Division of Reproduction and Obstetrics, School of Veterinary Medicine and Biomedical Sciences, IPB University, Bogor 16680, Indonesia; 2 Research Center for Applied Zoology, National Research and Innovation Agency (BRIN), Bogor 16911, Indonesia; 3 Department of Biology, Faculty of Science, IPB University, 16680, Bogor, Indonesia

**Keywords:** Fertility Markers, Heat Shock Protein 70-2, Madura Bulls, Protamine 1, Semen Quality

## Abstract

**Objective:**

This study aims to identify heat shock protein70-2 (HSP70-2) and protamine-1 (PRM1) mRNA and protein in Madura bull sperm and demonstrate their relation as bull fertility biomarkers.

**Methods:**

The Madura bull fertility rates were grouped based on the percentage of first service conception rate (%FSCR) as high fertility (HF) (79.04%; n = 4), and low fertility (LF) (65.84%; n = 4). mRNA of HSP70-2 and PRM1 with peptidylprolyl isomerase A (PPIA) as a housekeeping gene were determined by quantitative real-time polymerase chain reaction, while enzyme-linked immunoassay was used to measure protein abundance. In the post-thawed semen samples, sperm motility, viability, acrosome integrity, and sperm DNA fragmentation index were analyzed. Data analysis was performed on the measured parameters of semen quality, relative mRNA expression, and protein abundance of HSP70-2 and PRM1, among the bulls with various fertility levels (HF and LF) in a one-way analysis of variance analysis. The Pearson correlation was used to analyze the relationship between semen quality, mRNA, proteins, and fertility rate.

**Results:**

Relative mRNA expression and protein abundance of HSP70-2 and PRM1 were detected and were found to be highly expressed in bulls with HF (p<0.05) and were associated with several parameters of semen quality.

**Conclusion:**

HSP70-2 and PRM1 mRNA and protein molecules have great potential to serve as molecular markers for determining bull fertility.

## INTRODUCTION

Madura cattle, which have various advantages and great potential for breeding are local Indonesian beef cattle derived from crosses of *Bos indicus* and *Bos javanicus* [1]. However, Zuhri et al [2] reported that the results of the reproductive efficiency of Madura cattle are lacking; with percentage of conception rate (CR) currently below 60%, and decreasing population resulting from a high rate of slaughter of productive cattle. Thus, a cost-effective development program is urgently required to increase the population of this cattle breed. The artificial insemination (AI) program, widely believed to increase the population and reproductive efficiency of Madura cattle [3], is not optimal. Physical examination and certain sperm quality, the two parameters used to determine the fertility of superior bulls used for the AI program, are not accurate enough [4]. Recent studies report that 13,000 RNA and protein molecules, which are present in the sperm, influence the process of fertilization, early embryonic development, and even the health and phenotype of offspring and are more reliable in the accurate determination of male fertility [5]. The presence of irregularities or abnormalities in these sperm molecules significantly affects the ability of the sperms to fertilize oocytes and can interfere with embryonic development [6].

Heat shock protein70-2 (HSP70-2) and protamine-1 (PRM1) are known to play active roles in fertilization. Heat shock protein, a group of proteins with abundant chaperones, plays a role in protecting cells from apoptosis and oxidative stress [7]. Protamine, which is a core protein of the sperm, is rich in arginine, and plays a role in packaging sperm DNA by making it denser and protecting the DNA from various damaging factors [8]. HSP70-2 is widely distributed in the head, mitochondria, and tail of the sperm. Sahoo et al [9] reported that HSP70-2 and PRM1 mRNAs are important for normal sperm function and are associated with decreased motility and fertility. Moreover, HSP70-2 has also been reported to play an essential role in human spermatogenesis and sperm-egg recognition [7]. The significance of HSP70-2 and PRM1 in sperms has been widely studied and is reported to be closely associated with male fertility [8,10]. To date, no comprehensive studies of HSP70-2 and PRM-1 in bull spermatozoa at both the mRNA and protein levels, and the effect of these parameters on semen quality and fertility, have been reported for Indonesian bull breeds, especially Madura bulls.

Fertilization being a complex process, no single test or attribute can identify and distinguish bulls as being highly fertile or infertile. Due to suboptimal sensitivity and specificity, tests diagnosing fertility based on a single feature or marker may be less effective. The trend of identifying the most important fertility markers and combining the results to improve diagnostic accuracy is gaining precedence. Therefore, the characterization of potential genes and proteins can help to comprehensively understand the molecular basis and associations between sperm quality and fertilization, further enabling the identification of these genes as potential fertility biomarkers. Therefore, in this study, we identified and analyzed mRNA and protein expression levels of HSP70-2 and PRM1 in post-thawing sperm in Madura bulls and examined their association with fertility and semen quality. The results of this study are expected to reinforce the potential of HSP70-2 and PRM1 as prospective fertility biomarkers and, can be influential in increasing the population and genetic quality of native Indonesian, Madura cattle.

## MATERIALS AND METHODS

### Experimental animals

Frozen semen samples, collected during the dry season, which spanned from May to October from Madura bulls, maintainedble at Lembang AI Center, West Java, Indonesia, and Singosari AI Center, East Java, Indonesia were used for this study. Temperatures at these centers during semen collection ranged between 15°C to 30°C. One limitation of this study is that only eight Madura bulls from both AI Centers belonged to the productive age group (4 to 8 years old). These eight Madura bulls were classified based on the percentage of first service CR (%FSCR). The %FSCR assessment uses data from the National Animal Health Information System (iSIKHNAS), Indonesia, particularly in East Java, from 2018 to 2020. iSIKHNAS is a nationally integrated system that contains data on AI services and pregnancy examination data. It also includes data on bulls used for AI across all regions in Indonesia. Based on the data obtained from iSIKHNAS, the total number of inseminated Madura cows was 1,054. As previously reported, at least 100 insemination records are required for determining a statistically adequate %FSCR for each bull [19]. In accordance with the study conducted by Aslam et al [11], the impact of environmental factors, management practices, and cow-related factors on bull fertility were assumed to be negligible, since the focus was primarily on conducting numerous inseminations for determining the field CRs. The bull fertility was classified based on the %FSCR. The %FSCR for each bull was charted, and the mean and standard deviation (SD) were computed. The %FSCRs below “mean–1 SD” were used to identify low fertility (LF) bulls, whereas rates above “mean+1 SD” were used to identify high fertility (HF) bulls [4]. The LF bulls (n = 4) had a %FSCR of 65.84%±4.43%, while for HF bulls (n = 4), the %FSCR was 79.04%±5.80%. The fertility level classification of Madura bulls is indicated in Table 1.

### Frozen-thawed sperm quality

The quality analysis was carried out on frozen-thawed sperm in this study. Assessment parameters included sperm motility, viability, the integrity of acrosome, and DNA fragmentation index (DFI). The frozen sperm straws were thawed for 30 seconds at 37°C and then were immediately analyzed to determine each parameter. Five replicate straws for each bull were used in this study. Computer assisted sperm analysis (Minitüb, Tiefenbach, Germany) with SpermvisionTM 3.7 programs were used to evaluate the total and progressive motility of sperm, in accordance with the method described by Sundararaman et al [12]. Sperm movement patterns were observed using a 20× magnification objective lens with four fields of view, and the number of sperm cells ranged from 50 to 250 in each field of view (used for a microscope with a 0.63× video adapter). The viability of the sperm was assessed by forming a smear on slides from a mixture of 10 μL semen and 10 μL eosin-nigrosine (1:1) stain (0.82 g eosin Y, 5 g nigrosine, 0.375 g Na Citrate, and 100 mL aquadest), determined by. The slides were then dried on a heating table. Live sperm remained colorless (transparent), while dead sperms were stained red. 200 sperm cells were analyzed using a 40× magnification microscope in 10 fields of view [4].

The integrity of the sperm acrosome was analyzed using fluorescein isothiocyanate conjugated peanut agglutinin and propidium iodide (FITC-PNA and PI; Sigma Aldrich, Germany). Semen samples were air-dried at room temperature, followed by fixation in 96% ethanol at room temperature for 10 minutes and air-drying. Up to 30 μL of the 100 μg/mL PNA lectin solution was poured onto the smear sample followed by incubation at 37°C for 30 minutes. The smear sample was then incubated for 5 minutes with 5 μL of 1 μg/μL PI solution (Sigma, St. Luis MO, USA), after which it was washed three times with phosphate buffer saline (PBS) to eliminate any unbound reagent residue. The slide was then covered with a cover slip, and sperm acrosome integrity was evaluated by fluorescence microscopy (AxioPhot Zeiss, Oberkochen, Germany; 490/530 nm excitation filter) using a filter at 40× magnification. Acrosome-intact sperm showed a strong green acrosomal fluorescence cap, while sperm with damaged acrosome demonstrated a red fluorescence cap [13].

**Figure 1:**
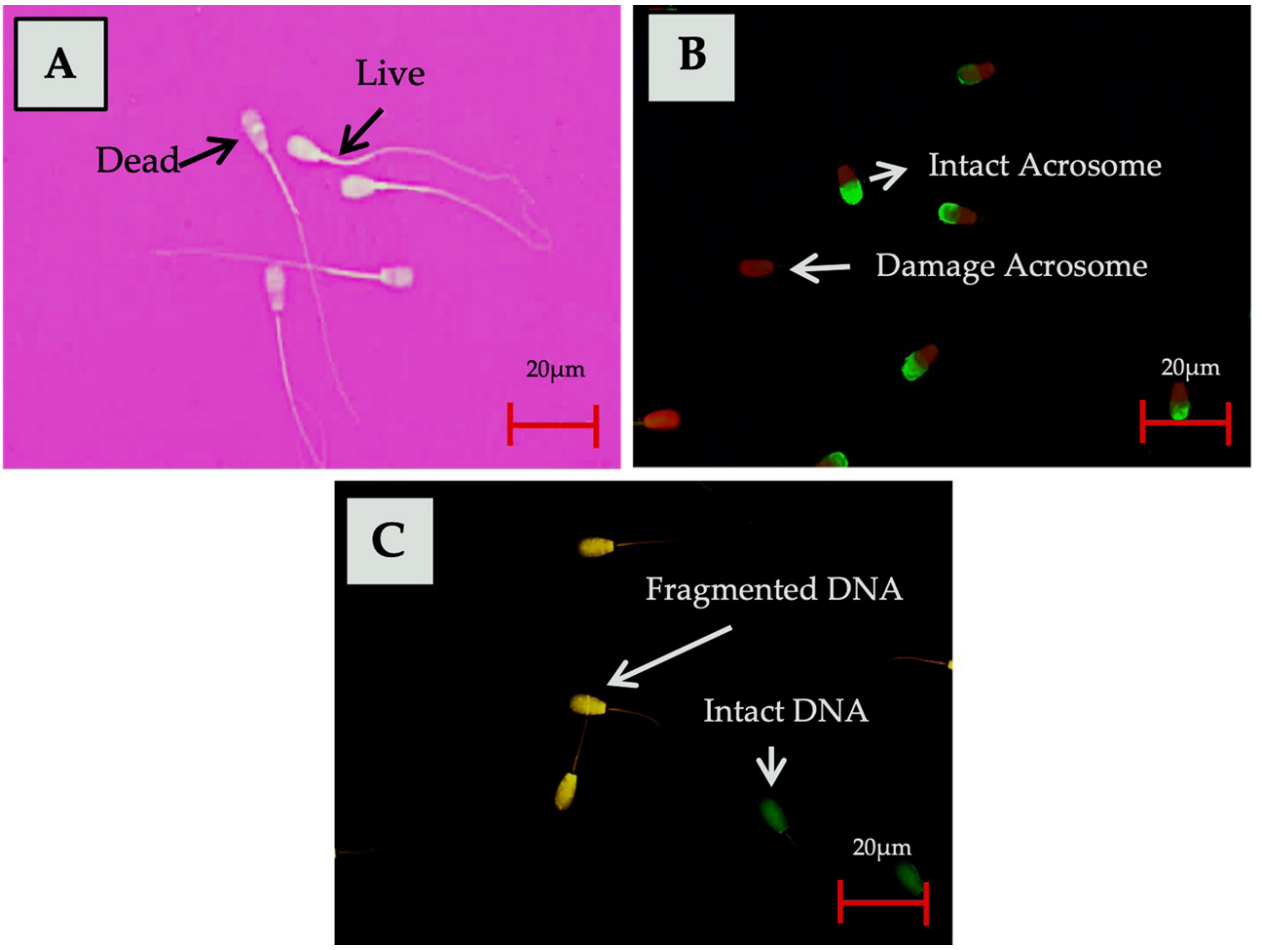
Assessment of sperm functional attributes. (A) Eosin-Nigrosin staining for viability assessment, (B) FITC-PNA staining to determine sperm acrosome integrity, (C) AO staining for detection of DNA integrity status. FITC-PNA, fluorescein isothiocyanate conjugated peanut agglutinin; AO, acridine orange.

Sperm DFI was assessed using acridine orange (AO) staining. A smear of 5 to 10 μL semen was fixed on a slide for two hours using Carnoy’s solution (methanol:glacial acetic acid [3:1]). The smear sample was dyed using 1% AO, 0.1 M citric acid, and 2.5 mL Na_2_O for 5 minutes in a dark environment. The slide was subsequently covered after washing specimen with distilled water. A total of 500 sperm cells were counted and evaluated under a fluorescent microscope (AxioPhot Zeiss, Germany; 490/530 nm excitation filter) using a filter at 400× magnification. Sperm cells with a normal DNA content exhibited green fluorescence, while those with fragmented DNA content exhibited a fluorescence that ranged from yellowish-green to reddish-orange [14]. The results of the analysis using the staining method on various semen quality parameters tested in this study are presented in Figure 1.

### Isolation and expression analysis of mRNA

Direct-zol RNA Miniprep Plus separated total RNA from post-thawed sperm (Zymo Research, Irvine, CA, USA). The sperm RNA solution was stored in the RNase-free tube at −80°C. RNA quality and quantity was assessed using Nanodrop (ND-1000; Thermo Scientific, Waltham, MA, USA). RNA samples with a 260/280 ratio of 1.8 to 2.0 were considered for cDNA synthesis. The cDNA was synthesized in accordance with the manufacturer’s protocol using the SensiFast cDNA synthesis kit (Meridian Bioscience (Bioline), Memphis, TN, USA). The cDNA synthesis procedure was carried out by adding several reactions with a total volume of 20 μL. The total volume of RNA used was 10 μL. The cDNA samples thus obtained were used for quantitative real-time polymerase chain reaction (qRT-PCR), analysis. The Sensifast SYBR mix (Thermo Scientific, USA) was used for the qRT-PCR analysis. The compositions used for the reactions included 1 μL each of forward and reversed primer, 10 μL of Sensifast SYBR mix, 6 μL of nuclease-free water, and 2 μL of cDNA stock sample. The qRT-PCR protocol was performed using CFX Opus 96 (Biorad, China) with an activation step of 95°C for 2 min. Amplification was carried out at a denaturation temperature of 95°C for 10 s for 40 cycles, annealing temperature according to the primer of each gene (Table 2) for 30 s. Each process was duplicated and included a non-template control. The resulting expression values were standardized to PPIA (peptidyl propyl isomerase A), the housekeeping gene. Schmittgen and Livak’s 2^−ΔΔCT^ technique was used for quantifying the qRT-PCR expression data for each gene [15].

### Sperm protein analysis

Each frozen sperm sample was thawed in a 37°C water bath for 30 seconds, washed with PBS and then centrifuged at 700×g for 15 minutes to pelletize it (approximately 25×10^6^ sperm cells/mL). The amount of HSP70-2 and PRM1 protein in the sperm pellets were then measured using the enzyme-linked immunoassay (ELISA) method, in accordance with the manufacturer’s instructions (Bovine HSP70-2: 2: Cat No. MBS2614143 and PRM1: Cat No. MBS2609702; MyBioSource, Inc., San Diego, CA, USA). Briefly, after preparation of the reagents, samples, and standards, the sperm pellets obtained after centrifugation were placed into the wells. The sperm pellet samples and each standard of bovine HSP70-2 and PRM1 were added to the respective reaction wells, which were then covered with adhesive tape and incubated at 37°C for 90 minutes. The anti-bovine HSP70-2 and PRM1 biotinylated antibody solutions were prepared 30 minutes before conducting the experiment. The EIA plate was washed twice, and 100 μL of antibody solution was added to the wells, which were then sealed with adhesive tape and incubated for 60 min at 37°C. The EIA plate was rinsed three times followed by the addition of 100 μL of an enzyme-conjugated solution that had been prepared 30 minutes earlier. The wells were then sealed and incubated at 37°C for 30 min. The EIA plate was then rinsed five times, the Color Reagent solution (100 μL) was added, and then the plate was incubated at 37°C for 30 minutes in the dark. In the final stage, 100 μL of Color Reagent C was added and was thoroughly mixed. In the end, an EIA reader was used to measure the number of HSP70-2 and PRM1 proteins in sperm with absorbance at 450 nm within 10 minutes.

### Statistical analysis

The data in this study were tested for normality and were normally distributed (p>0.05) based on the Kolmogorov–Smirnov test. Data analysis was performed on the measured parameters of semen quality, relative mRNA expression, and protein abundance of HSP70-2 and PRM1 among the bulls with various fertility levels (HF and LF) in a one-way analysis of variance analysis. In the event of a significant difference in each fertility group, Tukey’s post hoc test was applied. Pearson correlation was used for analyzing the relationship between semen quality, mRNA, proteins, and fertility rate. A linearity test was performed using Scatter Plot, for analyzing the relationship pattern between HSP70-2 and PRM1 mRNA expression and protein abundance with fertility rate (%FSCR). The data is presented in the form of mean±standard error. Statistical analysis was performed using statistical package for social sciences version 26 (IBM, NY, USA).

## RESULTS

The results of the quality analysis of the semen samples obtained from HF and LF Madura bulls are presented in Table 3. The mean percentages of total motility were 53.8%±1.7% and 50.8%±0.4% for HF and LF bulls, respectively. The mean percentages of progressive motility in HF and LF bulls were 47.6%±1.6% and 44.9%±1.2%, respectively (Table 3). HF bulls had a significantly higher proportion of total and progressively motile sperm (p<0.05). Meanwhile, the percentage of sperm viability in the post-thawed samples obtained from Madura bulls in the HF group was higher than LF (p>0.05). The mean percentages of sperm viability were 82.6%±1.5% and 72.5%±7.6% for HF and LF samples, respectively.

The mean percentages of acrosome-intact sperms were 83.1%±4.1% and 72.1%±6.7% in the HF and LF bulls respectively. The HF bulls had more acrosome-intact sperm than LF bulls (p<0.05). The mean percentage of sperm DFI in HF and LF bulls was 14.6%±1.3% and 17.2%±0.8%, respectively. However, LF bulls had a larger percentage of DFI (p<0.05) than HF bulls.

The results indicated that semen quality parameters were associated with fertility rates and were negatively correlated with %DFI (Table 4). Sperm motility was positively correlated with fertility rate (total motility: r = 0.532 and progressive motility: r = 0.514) and was negatively correlated with sperm %DFI (total motility: r = −0.325; progressive motility: r = −0.295). Sperm viability was positively correlated with fertility rate (r = 0.760) and was negatively correlated with sperm %DFI (r = −0.387). Sperm acrosome integrity was positively correlated with fertility rate (r = 0.712) and negatively correlated with sperm %DFI.

**Figure 2:**
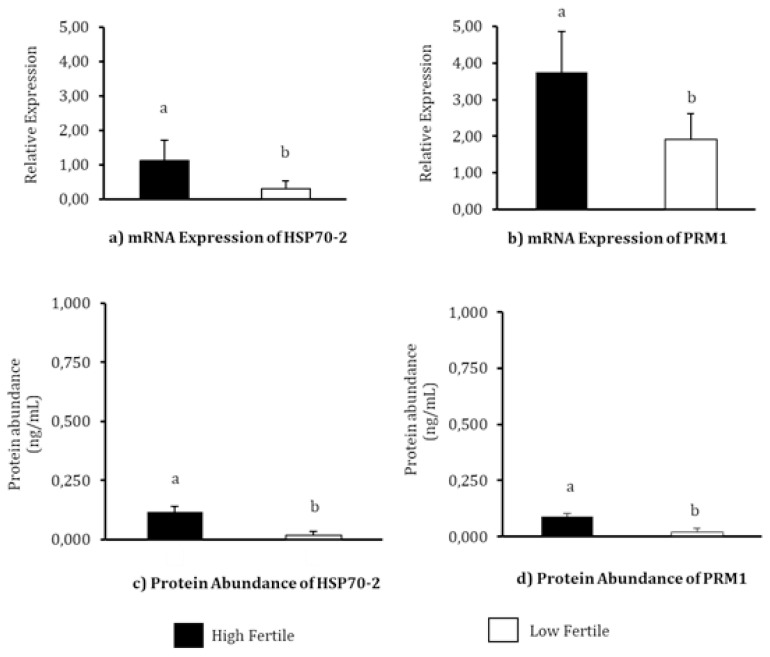
The mRNA relative expression and protein abundance of HSP70-2 and PRM1 in sperms of Madura bulls of three fertility groups. (a) mRNA expression of HSP70-2, (b) mRNA expression of PRM1, (c) Protein abundance of HSP70-2, and (d) Protein abundance of PRM1. HSP70-2, heat shock protein70-2; PRM1, protamine-1. ^a,b^ Different letters on each bar chart indicate significant differences (p<0.05) in each fertility group.

The relative mRNA expression and protein abundance of HSP70 and PRM1 in each fertility level has been depicted in Figure 2. The highest mRNA relative expression of HSP70-2 was observed in HF bulls (p<0.05). The mean mRNA relative expressions of HSP70-2 in HF and LF bulls were 1.12±0.6 and 0.29±0.24, respectively. The PRM1 also has the highest mRNA expression of PRM1 was also observed in HF bulls (p<0.05). The mean mRNA relative expression values observed in HF and LF bulls were 3.75±1.10 and 1.91±0.72, respectively. The results of HSP70-2 protein abundance analysis using EIA demonstrated a significantly high abundance of the protein in HF bulls (0.118±0.023 ng/mL, p<0.05) compared to LF bulls (0.018±0.017 ng/mL). The protein abundance of PRM1 in the Madura bull group was significantly different in the different fertility levels (p<0.05), with the highest concentration of PRM1 protein detected in HF group (0.089±0.013 ng/mL). The protein content of PRM1 in LF group was detected to be 0.020±0.017 ng/mL.

mRNA expression of HSP70-2 positively influenced the fertility levels (p<0.01), sperm total (p<0.01) and progressive (p<0.05) motility, viability (p<0.05), and acrosome integrity (p<0.01). Similarly, PRM1 mRNA expression also positively influenced the fertility levels (p<0.01), sperm total (p<0.05) and progressive (p<0.01) motility as well as acrosome integrity (p<0.05). At the protein level, HSP70-2 had a positive correlation with fertility levels (p<0.01), sperm total (p<0.01), and progressive (p < 0.05) motility. PRM1 protein abundance, on the other hand, had a positive correlation with fertility levels (p<0.01), sperm total motility (p<0.05), viability (p< 0.05), and acrosome integrity (p<0.01). HSP70-2 mRNA expression and its protein abundance as well as PRM1 mRNA expression and its protein abundance were found to be with DFI (p<0.05; Table 5).

**Figure 3:**
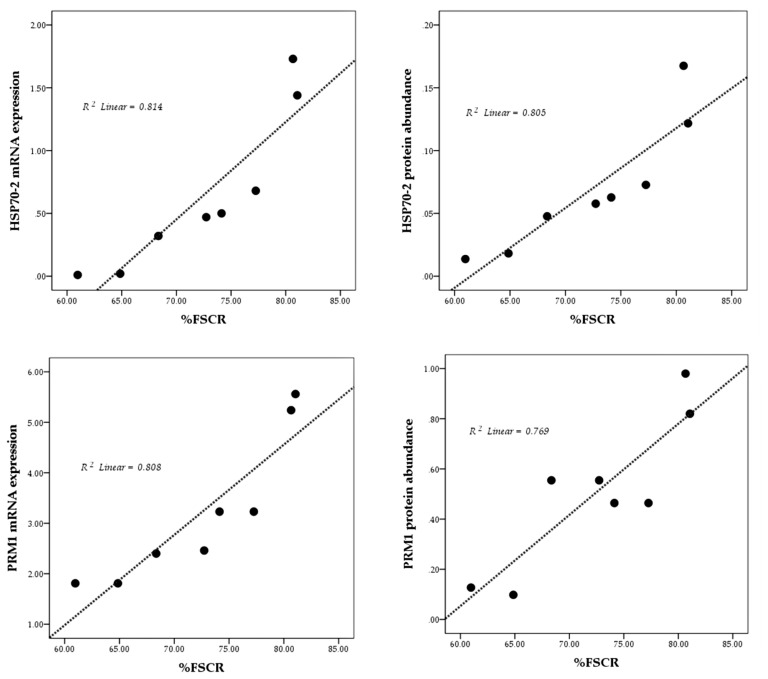
The linearity pattern (dotted line) of HSP70-2 and PRM1 mRNA expression and protein abundance with fertility rate (%FSCR). HSP70-2, heat shock protein70-2; PRM1, protamine-1; %FSCR, percentage of first service conception rate.

The results of the linearity test are presented in Figure 3 using scatterplots. Overall, mRNA expression and protein abundance of both HSP70-2 and PRM1 show a positive and linear relationship with fertility rate (%FSCR).

## DISCUSSION

Fertility of bulls is one of the determining factors for the success of AI programs. Semen quality plays an important role in determining the success of AI [6]. Frozen semen samples in an AI program are expected to have viable and motile sperms, capable of reaching the fertilization site, with membrane integrity for the formation of a sperm-oviduct reservoir [3,16], as well as an intact acrosome for the oocyte penetration process [4]. Pardede et al [8] stated that sperm DNA integrity also affects the fertility rate of bulls. Overall, the results of this study demonstrated that the percentage of good sperm characteristics fulfilled the requirements applied to the minimum standard of frozen semen for the AI program (progressive motility above 40%). Thus, there were no significant factors during the production and storage phases, that could potentially negatively influence the quality of the frozen-thawed semen, and that both national AICs manage bulls, freezing, and container systems adequately. Bahmid et al [17] claimed that frozen semen samples that had been preserved for 25 years, met the Indonesian national standard for AI. Sperm motility was also found to be positively correlated with the fertility rate in this study (p<0.01). Pardede et al [16] had also reported similar results, in which sperm motility was closely related to bull fertility. Only sperms with progressive motility movement successfully reached the site of fertilization.

During analysis of sperm viability, the percentage of damaged sperm membranes was found to be higher in LF bulls than in HF bulls, indicating reduced resistance of sperm to external factors in LF bulls. Acrosome integrity was also significantly lower (p<0.05) in LF bulls, thereby demonstrating a close relationship (p<0.01) between acrosome integrity and fertility. This is in agreement with a report by Pardede et al [16], according to which, an intact sperm acrosome is necessary for the process of capacitation, acrosome reaction, and oocyte fertilization making the integrity of the acrosome vital for fertility [18]. A significantly higher proportion of fragmented sperm DNA (p<0.05) was found in LF bulls and was strongly associated (p<0.01) with reduced fertility. Similar findings in which a significant relationship was observed between higher levels of sperm DNA fragmentation and a subsequent decrease in bull fertility have also been reported previously [19].

Overall, even though the semen quality parameters in this study were within a reasonable acceptable range and were classified as “good,” and had even met the specified requirements (progressive motility value above 40%), it was evident that this was not sufficient in assessing the fertility level of bulls. According to a previous report by Butler et al [20], conventional assessment of semen quality could not be considered sufficient to determine the fertility of bulls. Further studies involving a molecular approach need to be carried out to review various sperm functions that are genetic and cannot be explained solely on the basis of conventional investigations.

Heat shock protein member 2 and PRM1 are known to play an active role in fertilization [10,21,22]. HSP70-2 and PRM1 molecules in sperm have the potential to be used as molecular biomarkers for the assessment and differentiation of fertility levels in bulls. In the present study, HSP70-2 and PRM1 were identified in both HF as well as LF bulls’ sperm (Figure 2). Meanwhile, at the mRNA and protein levels, the abundances of HSP70-2 (Figure 2A, 2C) and PRM1 (Figure 2B, 2D) were found to vary significantly (p<0.05) between the three bull fertility levels, as evident in this study.

Heat shock protein70-2 is a molecule that is adjacent to ubiquitin in sperms. It is involved in spermatogenesis, protein binding, metabolism, and catalytic activity related to sperm function, and hence is critical in the process of fertilization. It not only impacts the success of fertilization, but also plays a role in the early development of the embryo [23]. Meanwhile, PRM1 is a molecule that is found in the sperm nucleus and performs the function of wrapping and protecting the DNA. PRM1 plays an important role in fertilization because it is associated with molecular defects in sperm during sperm maturation and affects fertility rates. Varying abundance of PRM1 in sperms is also associated with bull fertility [24]. However, the results of this study also demonstrated that the relative mRNA expressions and protein abundance of HSP70-2 and PRM1 were significantly (p<0.01) correlated and closely related to bull fertility (Table 5).

In general, HSP70-2 functions by increasing Ca^2+^-ATPase activity, reducing membrane damage, and increasing mitochondrial function. In addition, HSP70 stimulates superoxide dismutase activity and protects sperm from further oxidative damage. The HSP family is also reported to have a higher cellular tolerance to various types of stress that might have a damaging effect on cells. Thus, the HSP70-2 molecule protects mitochondria and intracellular sperm from stressors and is associated with the normal function of sperm motility in bulls [25]. Chen et al [26] have previously reported that the mRNA for HSP70 is involved in biological functions, especially in energy metabolism processes for sperm motility. Our study has also demonstrated a strong correlation between HSP70-2 and motility (p<0.01) (Table 5), thus reinforcing the hypothesis put forth by Chen et al [26] that, part addition to being involved in energy metabolism processes, HSP70 also prevents apoptosis due to heat stress and modulates the balance between dead and living cells [7]. Ramesha et al [27] also reported that HSP70, which plays a role in sperm-oocyte recognition, is found on the surface of the sperm plasma membrane, in the acrosome region, and is also detected during the capacitation process. These reports corroborate with our findings that HSP70-2 is correlated with sperm viability (p<0.05) and sperm acrosome (p<0.01) (Table 5). Recent studies on post-meiotic germ cells have revealed that HSP70-2 molecules are associated with nuclear transition proteins and are involved in packaging spermatid DNA [28]. These findings imply that HSP70-2 is necessary for controlling chromatin remodeling in spermatids. The absence of HSP70-2 in sperm may result in a lesser density of chromatin in the sperm nucleus, which can cause cell damage. Although not significant, the findings of this study seem to corroborate the reports indicating a negative correlation between HSP70-2 and the %DFI (Table 5).

mRNA expression and protein abundance of PRM1 were significantly higher (p<0.05) in HF bulls (Figures 2B, 2D) and is strongly associated with progressive motility (p<0.01) (Table 5). Previous studies have also indicated that PRM1 is closely related to progressive sperm motility in bulls [26]. Schneider et al [29] demonstrated that a decrease in Ca^2+^ in sperms with PRM deficiency results in the degradation of the plasma membrane, reduced sperm motility, and greater DNA damage. A high %DFI was closely related to low mRNA expression, and an abundance of PRM1 protein (p<0.05) (Table 5). As the main protein in the sperm nucleus of bulls, the arginine-rich PRM1 is required to condense the paternal genome and protect sperm DNA [29]. Thus, this study further reinforces existing research according to which PRM1 is negatively correlated with DFI percentage (p<0.05) (Table 5). According to Pardede et al [19], %DFI is inversely proportional to fertility, as evident from the poor CR in inseminated cows. Thus, this study proves the potential of mRNA expression and protein abundance of HSP70-2 and PRM1 as candidates functioning as accurate molecular markers for assessing bull fertility. This finding is further supported by plotting the relationship pattern between the relative mRNA expression and protein abundance of HSP70-2 and PRM1 with %FSCR (Figure 3). The positive linear relation supports the close association between each of these variables. In other words, HSP70-2 and PRM1, both at the mRNA and protein levels, are directly involved in the reproductive process, especially in the processes related to fertilization. Therefore, it is analysis of gene expression and protein abundance in sperms as potential fertility marker candidates and their effect on sperm functional traits is warranted. However, the relative expression of lower mRNA and proteins, especially in this case HSP70-2 and PRM1, was found to possibly interfere with sperm cellular function, thereby reducing the fertility performance of bulls.

Nonetheless, further studies are warranted to further strengthen this hypothesis and the findings of this study regarding the potential of HSP70-2 and PRM1 as potential biomarkers for predicting bull fertility. Research using a similar experimental design and carried out on other bull breeds will significantly enrich the results of this study. Considering that this research was carried out only on Madura bull sperm, anticipation of the influence of breed factors on the research results would be necessary. However, a biomarker would be expected to be applicable to all bull breeds and not just one bull breed. In addition, investigation on the association of HSP70-2 and PRM1 on the ability of sperm-oocyte fertilization, especially *in vitro*, is needed to further validate the results of this research. A study of the potential of HSP70-2 and PRM1 using bulls of different age groups, as well as productive and non-productive bulls, mainly related to standardization and determination of the abundance of HSP70-2 and PRM1 contained in frozen-thawed sperm needed by a bull to be categorized as a bull with HF will further strengthen the results of this research. However, this study supports the categorization of HSP70-2 and PRM1 as two of the many genes or proteins in sperms, that have great potential to be developed as biomarkers to accurately assess the fertility of bulls.

## CONCLUSION

In this study, we have revealed the association between mRNA expression and protein abundance of HSP70-2 and PRM1 in varying fertilities of Madura bulls as indigenous cattle in Indonesia. Relative mRNA expression and protein abundance of HSP70-2 and PRM1 were identified and were found to be abundant in bulls with HF and were associated with several parameters of semen quality. Each parameter of semen quality was seen to exist at a lower percentage in bulls with low fertility in this study. Semen quality parameters have a close relationship with the level of fertility. HSP70-2 and PRM1 mRNA and protein molecules have great potential to serve as molecular markers for determining bull fertility.

## Figures and Tables

**Table 1:** Classification of Madura Bull fertility^1)^

Bulls	%FSCR	Fertility level
Pajudan	82.77±0.19	HF
Montehai	81.76±1.41	HF
Pasean	81.26±1. 01	HF
Lombang	70.38±0.12	HF
Mangar	70.19±1.46	LF
Manding	69.13±0.68	LF
Siring	62.15±0.87	LF
Jengka	61.87±1.02	LF

1) Mean±standard deviation = 72.44±8.53.

%FSCR, percentage of first service conception rate; HF, high fertile (79.04±5.80); LF, low fertile (65.84±4.43).

**Table 2:** Primer used for quantitative real-time polymerase chain reaction

Genes	Primer sequence	Product size (bp)	Annealing temperature (°C)	Accession number
*HSP70-2*	F: TTGGGGACAAGTCAGAGAATG	118	53	NM_174344.1
	R: ATCGTGGTGTTCCTTTTGATG			
*PRM1*	F: AGATACCGATGCTGCCTCAC	234 (334 with intron)	53	NM_174156.2
	R: GTGGCATGTTCAAGATGTGG			
*PPIA*	F: ATGCTGGCCCCAACACAA	100	55	XM_001252921.1
	R: CCCTCTTTCACCTTGCCAAA			

*HSP70-2*, heat shock protein70-2; *PRM1*, protamine-1; *PPIA*, peptidylprolyl isomerase A.

**Table 3:** Sperm quality characteristics of Madura bulls from different fertility levels

Sperm characteristic	HF	LF
Total motility (%)	53.8±1.74^a^	50.8±0.4^b^
Progressive motility (%)	47.6±1.6^a^	44.9±1.1^b^
Viability (%)	82.6±1.5	72.5±7.6
Acrosome intact (%)	83.1±4.1^a^	72.1±6.7^b^
DNA fragmentation index (%)	14.5±1.3^a^	17.2±0.8^b^

HF, high fertile (79.04±5.80); LF, low fertile (65.84±4.43).

a,b Different letters indicate significant differences (p<0.05) in each fertility group.

**Table 4:** Correlation of functional sperm attributes with various fertility classifications

Traits	Fertility	TM	PM	Viability	Acrosome	DFI
Fertility	1	0.532^**^	0.514^**^	0.760^**^	0.712^**^	−0.455^**^
TM		1	0.780^**^	0.192	0.472^**^	−0.325^*^
PM			1	0.161	0.451^**^	−0.295
Acrosome					1	−0.171
DFI						1

TM, total motility; PM, progressive motility; DFI, DNA fragmentation index.

* p<0.05,

** p<0.01.

**Table 5:** Correlation of HSP70-2 and PRM1 mRNA expression and protein abundance with semen quality parameters and fertility levels

Item		TM	PM	Viability	Acrosome	DFI	Fertility
mRNA	HSP 70-2	0.885^**^	0.813^*^	0.765^*^	0.903^**^	−0.680	0.949^**^
	PRM1	0.731^*^	0.858^**^	0.641	0.791^*^	−0.786^*^	0.905^**^
Protein	HSP 70-2	0.999^**^	0.879^*^	0.629	0.739	−0.711	0.901^**^
	PRM1	0.854^*^	0.641	0.902^*^	0.924^**^	−0.852^*^	0.986^**^

HSP70-2, Heat shock protein70-2; PRM1, protamine-1; TM, total motility; PM, progressive motility; DFI, DNA fragmentation index.

* p<0.05,

** p<0.01.
